# Cytotoxicity of the Urokinase-Plasminogen Activator Inhibitor Carbamimidothioic Acid (4-Boronophenyl) Methyl Ester Hydrobromide (BC-11) on Triple-Negative MDA-MB231 Breast Cancer Cells

**DOI:** 10.3390/molecules20069879

**Published:** 2015-05-28

**Authors:** Alessandra Longo, Mariangela Librizzi, Irina S. Chuckowree, Christine B. Baltus, John Spencer, Claudio Luparello

**Affiliations:** 1 Dipartimento di Scienze e Tecnologie Biologiche, Chimiche e Farmaceutiche (STEBICEF), Edificio 16, Università di Palermo, Viale delle Scienze, 90128 Palermo, Italy; E-Mails: alessandra4682@hotmail.it (A.L.); merylib@alice.it (M.L.); 2 Department of Chemistry, School of Life Sciences, University of Sussex, Falmer, Brighton BN1 9QJ, UK; E-Mails: I.Chuckowree@sussex.ac.uk (I.S.C.); j.spencer@sussex.ac.uk (J.S.); 3 School of Science at Medway, University of Greenwich, Chatham ME4 4TB, UK; E-Mail: christine.baltus@univ-tours.fr

**Keywords:** boronic acid, BC-11, plasminogen activator inhibitor, breast cancer, cytotoxicity, MDA-MB231 cells

## Abstract

BC-11 is an easily synthesized simple thiouronium-substituted phenylboronic acid, which has been shown to be cytotoxic on triple negative MDA-MB231 breast cancer cells by inducing a perturbation of cell cycle when administered at a concentration equal to its ED_50_ at 72 h (117 μM). Exposure of cells to BC-11, either pre-absorbed with a soluble preparation of the N-terminal fragment of urokinase-plasminogen activator (uPa), or in co-treatment with two different EGFR inhibitors, indicated that: (i) BC-11 acts via binding to the N-terminus of the enzyme where uPa- and EGF receptor-recognizing sites are present, thereby abrogating the growth-sustaining effect resulting from receptor binding; and (ii) the co-presence of the EGFR inhibitor PD153035 potentiates BC-11’s cytotoxicity. Exposure of cells to a higher concentration of BC-11 corresponding to its ED_75_ at 72 h (250 μM) caused additional impairment of mitochondrial activity, the production of reactive oxygen species and promotion of apoptosis. Therefore, BC-11 treatment appears to show potential for the development of this class of compounds in the prevention and/or therapy of “aggressive” breast carcinoma.

## 1. Introduction

It is known that treatment of triple-negative breast cancer (TNBC) faces limited options due to the absence of expression of estrogen and progesterone receptors and human epidermal growth factor receptor 2 by neoplastic cells, which accounts for the dearth of targeted therapies, elevated tumor aggressiveness and poor patient prognosis [1]. To this end, a gamut of non-targeted treatment approaches have been investigated, including the use of anti-angiogenetic drugs, tyrosine kinase and PARP inhibitors. However, modest success has been achieved, also due to the marked heterogeneity of this neoplastic histotype [2]. Recently, attention has focused also on the critical role that may be played by the tumor microenvironment on TNBC invasive and metastatic aptitude and on the possibility of finding targets in the extracellular milieu which can be used to counteract TNBC progression (for example, see [3]).

**Figure 1:**
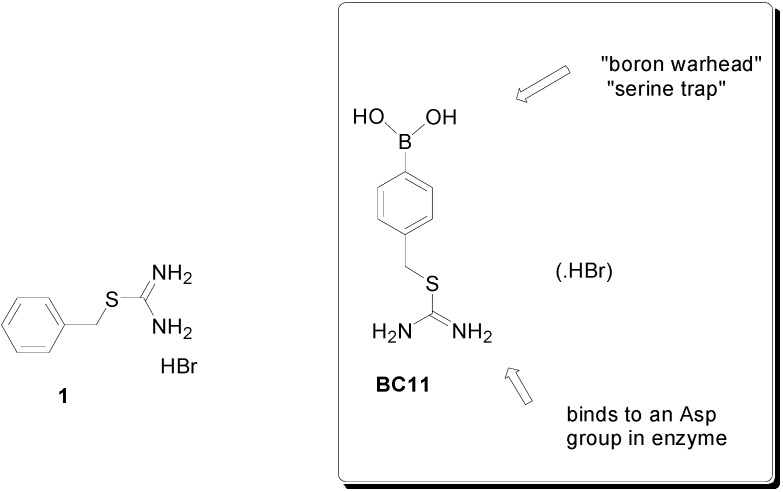
Thioronium salts in this study.

A great deal of experimental evidence have indicated that the over-expression of the extracellular serine protease urokinase-plasminogen activator (uPa) and its receptor uPAR actively contributes to the aggressive phenotype of a number of cancers, and anti-uPaR antagonistic antibodies have been proven successful in reducing TNBC growth *in vivo* [4]. Carbamimidothioic acid (4-boronophenyl)methyl ester hydrobromide (BC-11) is a thiouronium-substituted phenylboronic acid [5,6] originally synthesized as part of a chemical fragment library aimed at targeting thrombin and related serine protease enzymes and found to be a selective, single digit micromolar uPa inhibitor [7,8] (Figure 1). In this study, our goal was to comprehensively examine the effects of BC-11 on an “*in vitro*” model system of TNBC, *i.e.*, MDA-MB231 cells, with respect to viability and proliferation via a MTT assay, flow cytometric evaluation of cell cycle distribution, apoptosis modulation, and mitochondrial metabolic state.

The results indicate that BC-11 may be included in the list of drug-like molecules capable of inducing cytotoxicity on TNBC cells “*in vitro*”.

## 2. Results and Discussion

**Figure 2:**
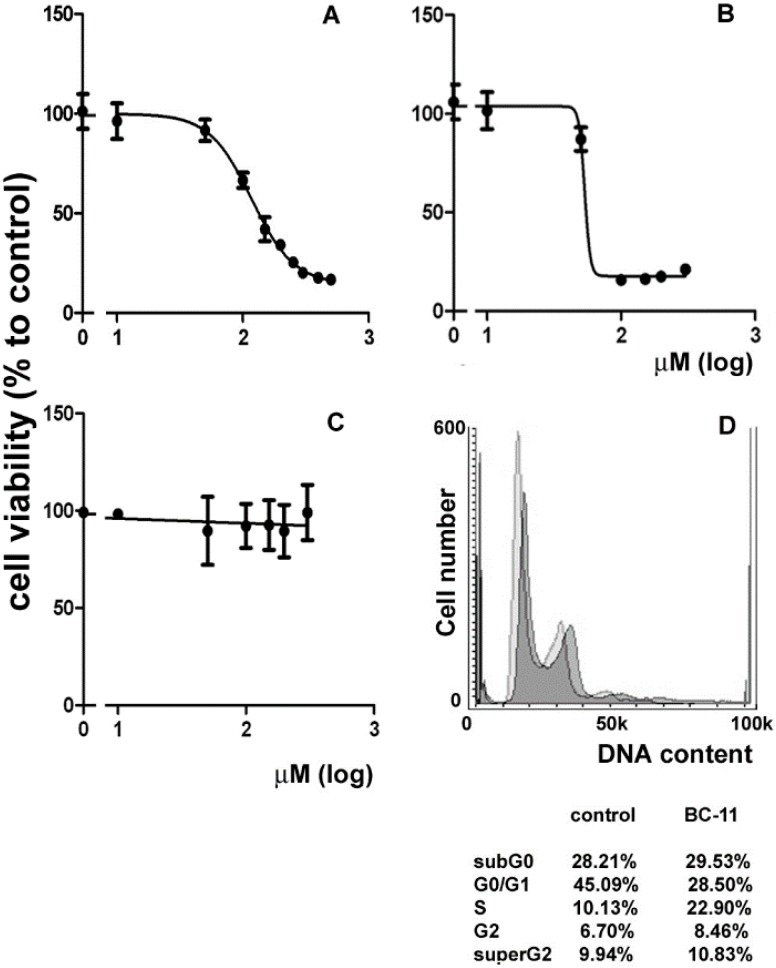
Effect of the different compounds on the viability of MDA-MB231 cells and of the exposure to BC-11 at its ED_50_ at 72 h on cell cycle. Dose-response curve for MDA-MB231 cell viability after treatment with (**A**) BC-11; (**B**) doxorubicin and (**C**) **1** for 72 h. The ED_50_ of BC-11 and doxorubicin are 117 and 80 μM, respectively. The ED_75_ of BC-11 is 250 μM. Error bars correspond to s.e.m. of three independent measurements; (**D**) DNA profiles of MDA-MB231 cells after 72 h of culture in control conditions (lighter in the background) and in the presence of 117 μM BC-11 (darker superimposed). Total cell distribution is reported in the annexed table.

In a first set of assays, we checked the effect of dose- and time-dependent incubation with BC-11 on MDA-MB231 cell survival via an MTT assay. As shown in Figure 2A, when cells were exposed for 72 h to BC-11, viability decreased in a concentration-dependent manner with a half maximal effective dose (ED_50_) = 117 μM (BC-11 can be considered to be a “rule of three” fragment, with expected relatively moderate activity [9]). Doxorubicin, a clinically-employed anti-TNBC chemotherapeutic used as a control in the MTT assay, also decreased cell viability in a concentration-dependent manner with around a 32% lower ED_50_ (80 μM) under the present experimental conditions (Figure 2B). Interestingly, when cells were exposed to (benzylsulfanyl)methanimidamide hydrobromide (**1**) [5], a BC-11 analogue lacking the essential boron functionality that leads to serine protease inactivation [10,11] (Figure 1), no effect on viability could be observed (Figure 2C) thereby confirming the implication of the enzyme-linking moiety in BC-11’s inhibitory role.

The ED_50_ concentration of BC-11 was chosen for the first group of experiments aimed to glean more detailed data on the molecular mechanism of BC-11-induced toxicity on MDA-MB231 cells via flow cytometry. No significant death induction was observed after 24 and 48 h of incubation (not shown). The results obtained indicated that no apoptosis induction at early (6 and 24 h) and late stages (72 h) as well as reactive oxygen species (ROS) production or mitochondrial transmembrane potential (MMP) dissipation at 72 h of exposure to BC-11 could be observed (not shown). Interestingly, when MDA-MB231 cells were tested for the distribution of cell cycle phases, as shown in Figure 2D, 72 h-exposure to BC-11 was linked with a decrease of the G_0_/G_1_ phase fraction (28.50% *vs.* 45.09%) and an increase of the S phase fraction (22.90% *vs.* 10.13%), indicative of a restrained progression through S phase conceivably due to the activation of the correspondent checkpoint.

It is known that the amino-terminal fragment (ATF; aminoacids 1–135) of the non-catalytic A chain of uPa contains an “EGF-like” and a “kringle” domain, the first one encompassing the uPa receptor (uPaR) binding site and able to exert growth factor-like effects, and the second one intervening in the stabilization of ligand-receptor binding [10,11]. The ability of the uPa-uPaR system to sustain growth and abrogate apoptosis of normal and neoplastic cells, including MDA-MB231, via modulation of signal transducers (such as PI3K/Akt and Ras/ERK) has been widely acknowledged (e.g., [12,13]). In addition, interaction of uPa with the EGF receptor (EGFR) has also been reported (e.g., [14,15,16]), and, due to the EGFR positivity of TNBC [17], it has been acknowledged that this breast cancer subtype might benefit from EGFR-targeted therapy (e.g., [18]). In light of the literature precedents, we therefore ascertained whether BC-11’s cytotoxic activity on MDA-MB231 cells, could be ascribed to its binding to the uPaR- and EGFR-recognizing site of the enzyme, thereby competing with the receptor(s) and switching-off the related proliferation and survival-promoting intracellular signalling pathways. To this purpose, we used the reversible tyrosine kinase, EGFR-inhibitors based on quinazolines, *N*-(3-bromophenyl)-6,7-dimethoxyquinazoline-4-amine (PD153035, IC_50_ of <30 pM *vs.* EGFR) thereafter named 3-B, and *N*-(4-bromophenyl)-6,7-dimethoxyquinazoline-4-amine, thereafter named 4-B. In this set of experiments, MTT assays were performed to monitor the viability of cells (i) exposed to BC-11 reacted with preparations of 100 or 500 ng or 1 μg of isolated ATF before administering to cell cultures; and (ii) co-treated with BC-11 and either or both of EGFR inhibitors to check the occurrence of a synergistic, antagonistic or additive effect of the compounds.

**Figure 3:**
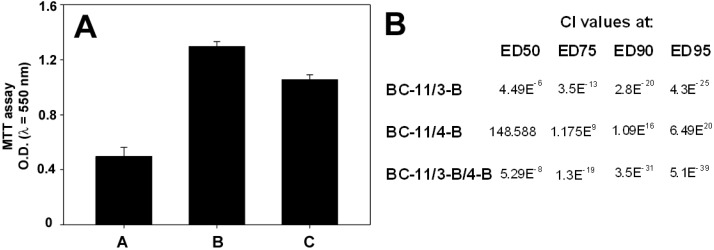
Effect of ATF, 3-B and 4-B on BC-11 cytotoxic activity. (**A**) MTT assay of MDA-MB231 cells treated with 117 μM BC-11 (A), untreated (B) and treated with 117 μM BC-11 pre-adsorbed with 100 ng ATF for 72 h. Error bars correspond to s.e.m. of three independent measurements; (**B**) CI values of BC-11 in two-drug and three-drug associations with the EGFR inhibitors 3-B and 4-B at different effect dose levels.

As shown in Figure 3A, pre-treatment of BC-11 with 100 ng ATF resulted in the reversion of BC-11 cytotoxicity, confirming its competitive effect towards drug binding to the uPa “growth-promoting” domain. Concerning the two EGFR inhibitors, in a pilot set of assays we first evaluated their individual effect towards MDA-MB231 cells after 72 h of exposure and demonstrated a dose-dependent impairment of cell viability with an ED_50_ = 35 μM for 3-B and an ED_50_ = 130 μM for 4-B (not shown). The table in Figure 2B reports the Combination Index (CI) of two-drug and three-drug co-treatments at constant ratios based on their ED_50_ values (3-B:4-B:BC-11 = 1:4:4). The data obtained point to opposing, strongly, antagonistic or synergistic effects of compounds 4-B and 3-B, respectively, when administered for 72 h to MDA-MB231 cells in association with BC-11 at a ratio based on similar efficacies. Interestingly, the synergistic effect of 3-B was maintained and even potentiated also in three-drug co-treatments.

**Figure 4:**
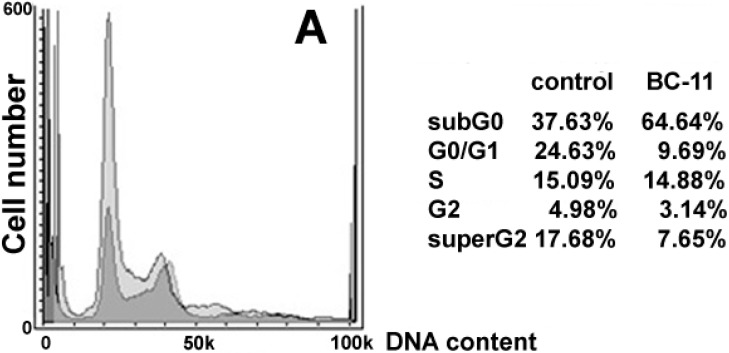

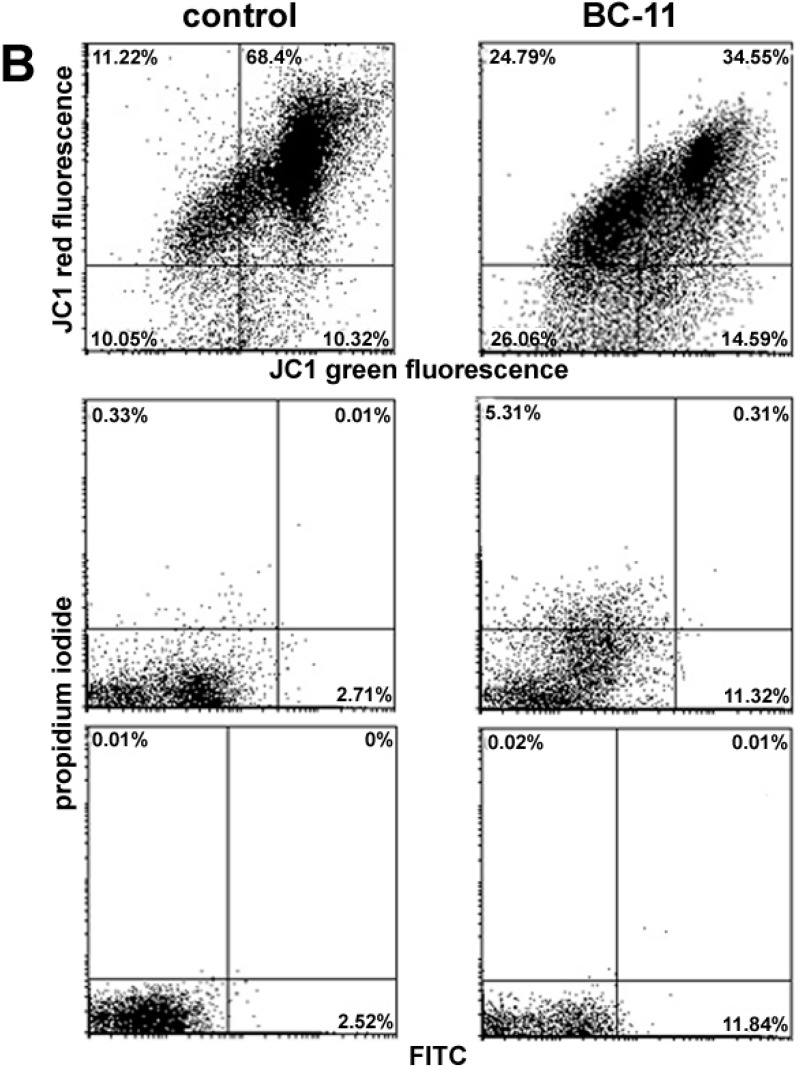
Effect of exposure of MDA-MB231 cells to BC-11 at its ED_75_ at 72 h on cell cycle, mitochondrial activity and apoptosis promotion. (**A**) DNA profiles of MDA-MB231 cells after 72 h of culture in control conditions (lighter in the background) and in the presence of 250 μM BC-11 (darker superimposed). Total cell distribution is reported in the annexed table; (**B**) Panel of flow cytometric assays of control cells and parallel cultures exposed to 250 μM BC-11. Analysis of MMP through JC-1 staining is reported in the top dot plots where the percentages indicated in the bottom quadrants in each frame refer to low red-emitting cells that underwent dissipation of MMP. The middle dot plots report the result of the staining with two-colour ROS detection reagents. The percentage indicated in the quadrants in each frame refers to superoxide only overproducing cells (top left quadrant), total ROS overproducing cells (bottom right quadrant), and total ROS/superoxide overproducing cells (top right quadrant). Analysis of phosphatydilserine externalization through annexin V-FITC coupled to propidium iodide (PI) staining is reported in the bottom dot plots. The percentage indicated in the quadrants in each frame refers to necrotic annexin^−^/PI^+^ and annexin^+^/PI^+^ cells (both top quadrants) and apoptotic annexin^+^/PI^−^ cells (bottom right quadrant).

In order to obtain a more complete characterization of BC-11’s biological activity on triple-negative breast cancer cells, a last set of experiments was performed by incubating MDA-MB231 cells with a higher concentration of the drug corresponding to its ED_75_ at 72 h (*i.e.*, 250 μM), and checking whether the enhanced cytotoxic effect could be due to the onset of additional death-inducing events (*i.e.*, apoptosis, MMP collapse, ROS hyperproduction), or only to a more pronounced uPa blocking effect following the increase in BC-11 concentration. As expected, cell cycle analysis of the exposed cell preparation (Figure 4A) showed a decrease of G_0_/G_1_ phase fraction (9.69% *vs.* 24.63%), although an effect on S phase fraction was not recorded in this case.

3-B is a known potent inhibitor of EGFR autophosphorylation that competes with the ATP binding site [19] and, although MDA-MB231 cells have been proven to be weakly sensitive to its effect if compared to other cancer cell lines [20], under the experimental conditions tested a greater than expected additive effect was observed. Noteworthy, exposure to 3-B was shown to suppress endogenous uPa secretion by MDA-MB231 cells [21] which display a markedly up-regulated expression of this gene [22].

Consequently, the synergistic effect might be ascribed to the drastic reduction of the amount of secreted uPa allowing BC-11 to block more efficiently the less concentrated binding sites of the enzyme released in the extracellular medium. No attempt was made to get more into mechanistic details of the opposite effects exerted by EGFR inhibitors 3-B and 4-B, which will be the object of future investigation.

In addition, treatment with 250 µM of BC-11 was linked to an augmented subG_0_ fraction (64.64% *vs.* 37.63%), consistent with an increased accumulation of damaged and fragmented cells due to compound toxicity. Differently from the results obtained with BC-11 administered at an ED_50_ dose, the increase up to 250 μM of the drug resulted in the additional impairment of mitochondrial activity. As shown in the top and middle dot plots of Figure 4B, BC-11 induces the doubling of the number of mitochondria displaying dissipation of MMP paralleled by enhanced production of ROS (11.32% *vs.* 2.71%) including the superoxide anion (5.31% *vs.* 0.33%). It is well-known that mitochondria are the major intracellular sources of ROS and that in these organelles also ROS detoxifying systems are actively operating in physiological conditions [23]. BC-11, therefore, is likely to induce an oxidative stress due to mitochondrial defence failure [24], such as structural impairment and/or loss of anti-oxidant matrix solutes (e.g., glutathione) through the permeability transition pores, leading to imbalance of ROS production, removal and extra-mitochondrial release and resulting in the increase of their net intracellular accumulation. Excess ROS-mediated mitochondrial permeabilization is also known to release factors triggering the intrinsic apoptotic cascade (e.g., [25]). This can be correlated with the results shown in the bottom dot plots of Figure 4B, which indicate that an increase from 2.52% up to 11.84% of cells showing annexin^+^/propidium iodide (PI)^−^ pattern, *i.e.*, in early apoptosis, can be observed after exposure to the higher concentration of BC-11.

## 3. Experimental Section

### 3.1. Cell Culture and Drugs

MDA-MB231 breast tumor cells (obtained from ATCC, Manassas, VA, USA) were maintained in RPMI 1640 medium plus 10% foetal calf serum, 100 U/mL penicillin, 100 µg/mL streptomycin, and 2.5 mg/L amphotericin B (Invitrogen, Carlsbad, CA, USA), at 37 °C in a 5% CO_2_ atmosphere. The cells were detached from flasks with 0.05% trypsin-EDTA, counted, and plated at the necessary density for treatment after achieving 60%–80% confluency.

Carbamimidothioic acid (4-boronophenyl)methyl ester hydrobromide (BC-11) was synthesized according to a known route or purchased from Tocris Biosciences (Bristol, UK) [26]. (Benzylsulfanyl)methanimidamide hydrobromide (**1**) was made using a literature route. Doxorubicin was purchased from Tocris Biosciences. Reversible tyrosine kinase, EGFR-inhibitors based on quinazolines, namely *N*-(3-bromophenyl)-6,7-dimethoxyquinazoline-4-amine and *N*-(4-bromophenyl)-6,7-dimethoxyquinazoline-4-amine, were made according to literature routes as tool compounds and as positive assay controls [27,28,29].

### 3.2. MTT Assay

Assessment of cell viability was determined by an MTT assay [30]. Briefly, MDA-MB231 cells in exponential growth were plated at a concentration of 5500 cells/well in a 96-well plate, allowed to adhere overnight, and then treated with: (i) different concentrations of BC-11 for 24, 48 and 72 h; (ii) different concentrations of **1** and doxorubicin for 72 h; (iii) 117 μM BC-11 pre-adsorbed overnight in the cold with 100 ng of uPa-ATF [31] for 72 h; or (iv) BC-11 in association with either or both 3-B and 4-B compounds for 72 h at different concentrations. After addition of MTT (final concentration 0.75 mg/mL) and incubation with the SDS-containing solubilization buffer, the absorbance of the dissolved formazan was measured in an automated microplate reader at 550 nm. Cell viability ratio was determined as the ratio between treated cells and controls and ED_50_/ED_75_ were calculated with Prism 5.0 software (GraphPad, La Jolla, CA, USA). The synergistic, additive or antagonistic interactions of co-treatment with BC-11 and EGFR inhibitors was evaluated calculating the Combination Index (CI) with CompuSyn software (ComboSyn Inc., Paramus, NJ, USA; [32]) taking into account that CIs < 1 indicate synergism, whereas CIs ≥ 1 indicate additive effect or antagonism, respectively.

### 3.3. Flow Cytometry

Flow cytometric assays were performed according to Librizzi *et al.* [30]. The occurrence of early apoptosis was evaluated using the Annexin V-FITC kit (Miltenyi Biotec GmbH, Bergisch Gladbach, Germany) according to manufacturer’s instructions. Data were represented as dot plots using Flowing Software v.1.6.0., which discriminate normal cells (bottom left quadrant) from cells in early apoptosis (bottom right quadrant), cells in late apoptosis or early necrosis (top right quadrant), or cells undergoing necrosis (top left quadrant).

The production of ROS, such as hydrogen peroxide, peroxynitrite, hydroxyl radicals, nitric oxide, and peroxy radical, and of superoxide was evaluated using the Total ROS/Superoxide Detection Kit (Enzo Life Sciences, Lausen, Switzerland) according to manufacturer’s instructions. Positive (pyocyanin-treated) and negative (*N*-acetyl-L-cysteine-treated) controls were included in the analysis, and data were represented as dot plots using Flowing Software v.1.6.0., which discriminate cells with increased total ROS production (top left quadrant), increased superoxide production (bottom right quadrant) and increased total ROS and superoxide production (top right quadrant).

MMP was checked using the fluorescent dye JC1 (Molecular Probes, Eugene, OR, USA) that is selectively taken up into mitochondria, undergoing to a fluorescence emission shift from green (~529 nm) to red (~590 nm) in case of intact MMP, whereas in case of mitochondrial depolarization a decrease in the red/green fluorescence intensity ratio can be observed. Data were represented as dot plots using Flowing Software v.1.6.0., which discriminate in the bottom quadrants the amount of cells that undergo to loss of MMP. Cell cycle distribution was checked by PI stain after pre-incubation with Triton X-100 and RNase A, and analyzed with Flowing Software v.1.6.0. software. All the preparations assayed contained both attached and floating cells, and all the analyses were performed in a FACSCanto apparatus (BD Biosciences, Franklin Lakes, NJ, USA).

## 4. Conclusions

The collective results on BC-11’s cytotoxic effect obtained in “*in vitro*” assays on TNBC MDA-MB231 cells represent promising preliminary data indicating that BC-11 treatment possesses the potential for the development of this chemical class as an agent for the prevention and/or therapy of “aggressive” breast carcinoma, thus prompting further and more detailed investigations to evaluate BC-11 as anti-cancer compound *in vivo* (for reviews on bioactive boronic acids see [33,34]).
